# Novel Immunotherapeutic Approaches for Neuroblastoma and Malignant Melanoma

**DOI:** 10.1155/2018/8097398

**Published:** 2018-10-30

**Authors:** Fabio Morandi, Francesco Frassoni, Mirco Ponzoni, Chiara Brignole

**Affiliations:** ^1^Stem Cell Laboratory and Cell Therapy Center, IRCCS Istituto Giannina Gaslini, 16148 Genova, Italy; ^2^Laboratory of Experimental Therapies in Oncology, IRCCS Istituto Giannina Gaslini, 16148 Genova, Italy

## Abstract

Neuroblastoma (NB) and malignant melanoma (MM), tumors of pediatric age and adulthood, respectively, share a common origin, both of them deriving from the neural crest cells. Although NB and MM have a different behavior, in respect to age of onset, primary tissue involvement and metastatic spread, the prognosis for high stage-affected patients is still poor, in spite of aggressive treatment strategies and the huge amount of new discovered biological knowledge. For these reasons researchers are continuously attempting to find out new treatment options, which in a near future could be translated to the clinical practice. In the last two decades, a strong effort has been spent in the field of translational research of immunotherapy which led to satisfactory results. Indeed, several immunotherapeutic clinical trials have been performed and some of them also resulted beneficial. Here, we summarize preclinical studies based on immunotherapeutic approaches applied in models of both NB and MM.

## 1. Background

Neuroblastoma (NB) and malignant melanoma (MM) share a common origin, arising from the neuroectodermal tissue, the portion of the ectoderm that gives rise to the central and peripheral nervous systems. In spite of this common feature, these tumors display a different behavior, in terms of both age of onset and tissues involvement. By one hand, NB is a pediatric tumor, with a median age at diagnosis of about 17 months, and only 10% of cases occurring in people older than 5 years of age [1]; on the other hand, MM usually affects adults, with an average age at diagnosis of 52 years [2].

Tissue involvement, clinical behavior, and metastatic spread are also strongly different. NB usually arises in the abdomen, and about 50% of cases present at diagnosis metastases at bone marrow (70.5%), skeleton (55.7%), lymph nodes (30.9%), liver (29.6%), or intracranial (18.2%) [1, 3, 4]. In contrast, MM arises from the malignant transformation of melanocytes in the skin, and preferentially metastatizes to lymph nodes and visceral sites (*i.e.*, lung and liver) [5].

In both tumors, the survival rate at 5 years for high-risk patients with metastatic disease is poor, in spite of the application of chemotherapy and aggressive treatment strategies [6–8]. For these reasons, novel therapeutic approaches are desperately needed and, in this view, immunotherapy represents a promising tool.

Immunotherapy lies on the possibility to exploit the host immune system to fight cancer [9, 10]. Two main strategies of cancer immunotherapy can be adopted: active and passive based on their ability to engage the host immune system to fight cancer. Moreover, both of them can be also classified in base of their antigen specificity [11, 12]. The active immunotherapy specifically consists in the stimulation of the patient's immune system, with the final aim to trigger an immune response against cancer cells, finally leading to their killing. Examples of active immunotherapeutic approaches are tumor vaccine and check point inhibitors, which work upon the engagement of the host immune system [13, 14]. On the other hand, passive immunotherapy makes use of the adoptive transfer of substances with immunomodulatory activity. Examples of passive immunotherapeutic strategies are represented by tumor-targeting monoclonal antibodies (mAb) and adoptive transfer of T cells which per se are endowed with antineoplastic activity [15, 16].

## 2. Neuroblastoma

NB is the most common extracranial solid tumor of pediatric age, accounting for about 15% of cancer-related deaths in children [1, 17, 18]. It is a highly heterogeneous disease, with several factors (*i.e.*, age at diagnosis, stage of the disease, genetic abnormalities, and instability) cooperating altogether to determine its behavioral progress [19]. In the last decade, many efforts have been spent to increase the knowledge about its cellular and molecular biology; nevertheless, this has not been translated in successfully improved treatment strategies. Indeed, although the application of aggressive treatment protocols consisting of surgery, intensive chemotherapy, radiation therapy and hematopoietic stem cell transplantation, the clinical outcome for patients affected by high-risk NB led to unsatisfactory results, being the overall survival around 40–50% [7, 20, 21].

Between the different approaches that have been studied to try ameliorate the clinical outcome of high-risk NB-affected patients, immunotherapy, mainly in the last two decades, has gained a lot of attention, also resulting in encouraging results [22].

### 2.1. Preclinical Studies on Neuroblastoma

Several preclinical studies have been ruled out, by testing different therapeutic approaches on NB animal models and different targets for immunotherapeutic protocols have been tested (Table 1). These therapeutic strategies are based on the employment of (1) immune effector cells, (2) monoclonal antibodies against tumor-associated antigens, (3) tumor vaccines, and finally (4) immunotherapeutic strategies aimed at harnessing the immune system as adjuvant for standard approaches.

#### 2.1.1. Cellular Therapies

Immunotherapeutic approaches can be based on the use of native or genetically modified immune effector cells that are able to recognize tumor-associated antigens, thus exerting specific cytotoxicity against tumor cells. These cells include the following: (1) engineered T cells specific for NB-associated antigens, (2) gamma delta T lymphocytes, and (3) cytotoxic T cells recognizing HLA-restricted tumor antigens and NK cells.

*Engineered T Cells Specific for GD2 and Other NB-Associated Antigens*. Disialoganglioside GD2 is expressed on tumors of neuroectodermal origin, including human NB and MM. The expression of this tumor-associated antigen is limited on normal tissues (it can be detected on cerebellum and peripheral nerves in humans), and this feature makes it an attractive target for antibody-based and cellular immunotherapy [23].

Different immunotherapeutic approaches for NB are based on chimeric antigen receptor- (CAR-) modified T cells specific for GD2 antigen. Richman et al. tested variants of CAR constructs to improve the stability and the affinity of the GD2 binding, and compared the properties of these variants in preclinical models of NB. Some of these mutations in the CAR construct enhanced antitumor activity against GD2^+^ human NB xenograft *in vivo.* However, this enhanced antitumor effect was associated with a CAR T cell infiltration and proliferation within the brain and neuronal destruction. This caused a lethal encephalitis localized to the cerebellum and basal regions of the brain, where GD2 is expressed at low levels. They concluded that GD2-specific CAR T cell therapy must be associated with additional strategies to control CAR T cell function within the central nervous system [24].

Since tumor-driven neoangiogenesis supports an immunosuppressive microenvironment that influences treatment responses, antiangiogenic drugs represent a promising therapeutic tool. Indeed, they promote infiltration of lymphocytes within the tumor by transiently reprogramming tumor vasculature. Thus, we investigated the anti-NB activity of GD2-specific CAR T cells combined with bevacizumab (BEV), a specific mAb against vascular endothelial growth factor (VEGFR), in an orthotopic xenograft model of human NB. We have demonstrated that GD2-CAR T cells displayed anti-NB activity only when combined with BEV, which did not inhibit tumor growth when administered alone. When combined with BEV, GD2-CAR T cells infiltrated tumor mass, where they secreted IFN-*γ* which, in turn, induced release of CXCL10 by NB cells. On the other hand, programmed cell death ligand (PD-L) 1 was upregulated on NB cells by IFN-*γ*, while tumor infiltrating GD2-CAR T cells expressed programmed death (PD) 1 inhibitory receptor. Thus, the PD-1/PD-L1 (molecules identified with negative immunoregulatory function) interaction may limit the antitumor efficacy of the combined treatment, and PD-L1 blocking strategies may further enhance the efficacy of such combination [25].

In another study, the authors developed GD2-specific CAR T cells using an anti-GD2 single-chain variable fragment (scFv) derived from a murine antibody of IgM class. This fragment was linked to the signaling domains of 4-1BB and CD3-ζ costimulatory molecules. These CAR T cells expressed high levels of anti-GD2 CAR, released TRAIL (TNF-related apoptosis inducing ligand) and IFN-*γ* upon cocultures with NB cells and exerted cytotoxicity against the latter cells. In a NB xenograft model, those GD2-specific CAR T cells infiltrated tumors and persisted into blood circulation, inducing apoptosis of NB cells and abrogating tumor growth [26].

At the present time, GD2-specfic CAR T cells have been tested in 9 clinical trials on NB patients: 3 of them are concluded, whereas 5 of them are still recruiting patients (http://www.clinicaltrials.gov).

NY-ESO-1 is a cancer-testis antigen expressed by different human solid tumors. Moreover, its expression on mature normal somatic tissues is very limited, thus suggesting that it may represent a promising target for tumor immunotherapy. Indeed, NY-ESO-1-specific engineered T cells have been recently successfully used in the treatment of adult tumors [27–29]. The expression of NY-ESO-1 has been demonstrated in 23% of primarily resected NB samples. T cells genetically modified to express an NY-ESO-1-directed high-affinity transgenic T cell receptor have been tested *in vitro* for their antigen specificity and anti-NB activity. Next, NY-ESO-1-targeted T cells have been tested in preclinical models of local and disseminated NB [30]. Anti-NY-ESO-1-specific T cells significantly delayed tumor progression and increased survival of mice bearing disseminated NB. These data suggested that NY-ESO-1 may represent a novel target for NB immunotherapy. Indeed, one clinical trial based on NY-ESO-1-specific CAR T cells is currently recruiting patients affected by NY-ESO-1 expressing malignancies, including NB (http://www.clinicaltrials.gov).

*Gamma Delta T Lymphocytes*. Gamma delta T lymphocytes (*γδ* T cells) display an innate cytotoxicity, which make them attractive for immunotherapy of cancer [31]. These cells recognize phosphoantigens, which represent natural nonpeptide phosphorylated intermediates of isoprenoid metabolism. These include exogenous prenyl pyrophosphates from bacteria and parasitic protozoa as well as endogenous prenyl pyrophosphates, *e.g*., isopentenyl pyrophosphate (IPP), deriving from the mevalonate pathway that operates in human cells [32]. Tumor cells produce elevated concentrations of IPP, and such production can be boosted by exposure to aminobisphosphonates, a class of drugs that inhibit osteoclastic resorption. Thus, tumor cells exposed to aminobisphosphonates can be recognized and killed by *γδ* T cells that recognize IPP and other prenyl pyrophosphates expressed on the surface of tumor cells [33]. Moreover, it has been shown that treatment with zoledronic acid (ZOL) combined with IL-2 activates and expands T cells expressing V*γ*9V*δ*2 T cell receptor (TCR) (V*δ*2^+^ T cells), the most common subset of *γδ* T cells. The latter cells are able to perform antibody-dependent cell-mediated cytotoxicity (ADCC) through expression of CD16 [34].

We have demonstrated in preclinical models of NB that the combined treatment with V*γ*9V*δ*2 lymphocytes and ZOL improved the overall survival of mice. This effect was achieved through the inhibition of tumor cell proliferation and angiogenesis and the induction of tumor cell apoptosis. Indeed, V*γ*9V*δ*2 T lymphocytes were attracted to NB-tumor masses of mice treated with ZOL, where they produced IFN-*γ* that, in turn, induced CXCL10 expression in NB cells. This study supports the use of V*γ*9V*δ*2 T lymphocytes as a therapeutic strategy for NB patients [35].

Another study showed that V*δ*2^+^ T cells exert ADCC against GD2^+^ NB cell lines, and such effect correlated with GD2 expression. In preclinical models of NB, the combination of adoptively transferred V*δ*2^+^ T cells (expanded *in vitro* with ZOL and IL-2) with anti-GD2 antibody ch14.18/CHO and with ZOL significantly suppressed tumor growth as compared to controls. Combination treatment was more effective than their use as single agents. This study delineates a possible use of this combined treatment as therapeutic strategy for GD2^+^ tumors, including NB [36].

Currently, three clinical trials based on ZOL for NB patients have been concluded, and one is still recruiting patients (http://www.clinicaltrials.gov).

*Cytotoxic T Lymphocytes and NK Cells*. PRAME (preferentially expressed antigen in melanoma) is a cancer-testis antigen, firstly discovered in a patient with melanoma, that is expressed by several solid tumors (*i.e.*, squamous cell lung carcinoma, medulloblastoma, renal cell carcinoma, and acute leukemia), including NB [37]. The limit in the employment of PRAME-specific HLA-class I-restricted cytotoxic T cells specific for antitumor immunotherapy is represented by the downregulation of HLA-class I molecules by tumor cells. This mechanism of immune evasion is adopted also by NB cells, which escape the recognition by NK cells and cytotoxic T lymphocytes (CTLs) through the downregulation of HLA-class I molecules and antigen presenting machinery components [38, 39].

Spel and colleagues have demonstrated that NB cells upregulated HLA-class I expression on their surface upon exposure to activated NK cells. Therefore, pretreatment with the latter cells sensitize NB cells to the recognition by specific CTLs, through the contact-dependent production of IFN-*γ*. Indeed, PRAME-SLLQHLIGL/HLA-A2-specific CTL clones specifically recognized HLA-A2^+^ NB cells. The authors concluded that NK cells may serve as adjuvant for CTL-based immunotherapy of NB [40].

Anaplastic lymphoma kinase (ALK) fusion proteins are oncogenic and are expressed in anaplastic large cell lymphomas (ALCLs) and other tumors, but not in normal tissues [41, 42]. Passoni and coworkers have demonstrated that ALK may represent a target for antigen-specific cell-mediated immunotherapy. A panel of ALK-derived peptides have been tested for their binding affinity to HLA-A^∗^0201 molecules and for their capacity to elicit a specific CTL-mediated antitumor immune response in preclinical models, using HLA-A^∗^0201 transgenic mice. They have demonstrated that HLA-A^∗^0201^+^ CTLs specific for the epitopes p280-89 (SLAMLDLLHV) and p375-86 (GVLLWEIFSL) are able to release IFN-*γ* upon stimulation with ALK peptide-pulsed HLA-matched cell lines. Moreover, these CTLs lysed HLA-matched ALCL and NB cell lines endogenously expressing ALK proteins. These results confirmed that ALK and ALK-derived peptides may represent suitable targets for the development of vaccination strategies [43].

In the last years, it has been demonstrated that NK cells are able to recognize and lyse NB cells through the recognition of ligands for natural cytotoxicity receptors (NCRs) expressed by tumor cells [44, 45]. However, NB cells express also ligands for NK cell inhibitory receptors, such as B7H3 [46]. The antitumor activity of NK cells in preclinical models of NB has been tested by Castriconi et al. who have administered polyclonal IL-2-activated NK cells to NB-bearing NOD/scid mice. They demonstrated that NK cells disseminated in different tissues, including those colonized by metastatic NB cells. Moreover, early repeated injection of NK cells in NB-bearing NOD/scid mice significantly increased their survival and reduced bone marrow infiltration. Finally, they showed that this therapeutic effect was further enhanced by administration of human recombinant IL-2 or IL-15 at low doses. These data suggested that NK-cell-based adoptive immunotherapy may represent a valuable adjuvant in the treatment of NB patients with metastatic disease, in a minimal residual disease setting [47]. Indeed, 14 clinical trials involving NB patients are currently based on NK cells infusion (4 completed, 4 active but not recruiting, and 6 active and still recruiting, http://www.clinicaltrials.gov).

#### 2.1.2. Monoclonal Antibodies

Several immunotherapeutic approaches are based on the use of specific mAbs against NB-associated antigens.

Anti-GD2 mAb ch14.18/CHO have been used in different preclinical studies [48]. This approach was also proven to be quite effective for treatment of high-risk NB patients, mainly through GD2-specific ADCC [49]. Different studies are aimed at enhancing the efficacy of the treatment with ch14.18/CHO ± IL-2. Siebert and colleagues have tested the combination of ch14.18/CHO-based immunotherapy with the blockade of PD-1 inhibitory receptor. Indeed, PD-1 is expressed by effector cells, while its ligand (PD-L1) is expressed by tumor cells, and upon this interaction, tumor cells are able to dampen antitumor immune response [50]. It has been demonstrated that upon 24 h of coculture with leukocytes and subtherapeutic concentrations of ch14.18/CHO, PD-L1 was strongly upregulated on NB cell lines. Such effect was further increased in the presence of IL-2, leading to the inhibition of ch14.18/CHO-mediated ADCC. ADCC was restored in the presence of nivolumab (anti-PD-1 mAb) or by preincubation with anti-CD11b mAb. Syngenic PD-L1^+^/GD2^+^ NB-bearing mice treated with ch14.18/CHO combined with anti-PD-1 mAb showed a strong reduction of tumor growth, prolonged survival, and the highest cytotoxicity against NB cells. These data suggested that the combination of ch14.18/CHO with PD-1 blockade may represent a new effective treatment strategy against GD2-positive cancers [51].

Tran et al. tested the combination of anti-GD2 antibody dinutuximab and galunisertib, an inhibitor of transforming growth factor beta 1 receptor (TGF*β*R1), on primary NB cells from patients. NB cells express mRNA of both transforming growth factor beta 1 (TGF*β*1) and its receptor. Engagement of TGF*β*R1 by TGF*β*1 activates SMAD proteins (which are the main signal transducers of TGF*β* superfamily) that translocate into the nucleus where they regulate gene transcription. Treatment with galunisertib suppressed activation of SMAD proteins induced in NB cells by exogenous TGF*β*1 or by plasma derived from patients' blood or bone marrow. Moreover, galunisertib suppressed SMAD2 phosphorylation in human NB cells growing in NSG mice. In addition Galunisertib, through the suppression of SMAD2 phosphorylation, restored the expression of DNAM-1, NKp30, and NKG2D cytotoxicity receptors and the TRAIL death ligand in NK cells, stimulated the release of perforin and granzyme A by the latter cells and enhanced NK cell-mediated cytotoxicity and ADCC against NB cells. Thus, the addition of galunisertib to adoptive cell therapy with NK cells plus dinutuximab reduced tumor growth and increased survival of NSG mice injected with NB cell lines or NB cells from patients [52].

Anti-PD-1 or anti-PD-L1 blocking mAbs have shown potent antitumor effects in adult cancer patients [53, 54]. Moreover, clinical studies have recently been started in pediatric cancers, including NB [55, 56]. Rigo et al. have shown that monotherapy with anti-PD-1/PD-L1 mAbs was not effective on systemic NB progression *in vivo* in two syngeneic models of disseminated NB, generated by the injection of Neuro2a or NXS2 cells, which express PD-L1. The combination of these mAbs with IL-21, POM-1 (inhibitor of ecto-nucleotidases), or anti-CD25 mAb was also ineffective in the same preclinical model. In contrast, the combination of anti-PD-1 or anti-PD-L1 mAbs with an anti-CD4 mAb resulted in a synergistic effect leading to significant increase of tumor-free survival of mice, complete tumor regression, and durable anti-NB immunity. Such effect was related to CD8-mediated cytotoxicity against tumor cells. The authors concluded that PD-1/PD-L1 and CD4^+^ regulatory T cells must be simultaneously blocked to achieve significant therapeutic effects [57].

Additional NB-associated antigens have been proposed in the last years as possible novel targets for immunotherapeutic protocols and tested in preclinical models of NB. CD171 (L1-CAM) is a cell surface adhesion molecule expressed at high levels on different solid human tumors that has been associated to tumor progression [58, 59]. The expression of CD171 has been detected also in NB and other tumors of the nervous system [60]. This molecule presents a glycosylation-dependent tumor-specific epitope that is recognized by the CE7 mAb. It has been demonstrated that CD171 is expressed in NB tumor specimens collected at diagnosis or relapse. Moreover, administration of CE7-CAR T cells displayed no toxicity in rhesus macaques and in a phase I study on NB patients, thus supporting their use for NB immunotherapy [61].

Recently, Bosse and coworkers have analyzed genes differentially expressed in NB and normal tissues, and they identified Glypican-2 (GPC2) as a molecule specifically expressed by NB cells and not by normal tissues. GPC2 expression has been detected on the cell surface in the majority of high-risk NB, and such expression correlated with worse prognosis of NB patients. Moreover, they have demonstrated that GPC2 expression is driven by somatic gain of chromosome 7q (where *GPC2* gene is localized) and by *MYCN* amplification. Finally, they developed a GPC2-directed antibody-drug conjugate, with a potent cytotoxic activity against GPC2-expressing NB cells. Thus, GPC2 may represent a promising immunotherapeutic target for NB patients [62].

At the present time, there are more than 60 clinical trials for NB patients based on the use of different mAbs, alone, or in combination with other therapeutic compounds (http://www.clinicaltrials.gov).

#### 2.1.3. Tumor Vaccines

Several therapeutic approaches have been performed using as tumor vaccines whole tumor cell lysates or irradiated tumor cells in combination or not with immune adjuvants.

A tumor vaccine that consist of irradiated tumor cells infected with the oncolytic IL-12-expressing HSV-1 virus, M002, has been tested in a NB preclinical model obtained by injecting Neuro-2a NB cell line in syngenic A/J mice. When mice were vaccinated with tumor vaccine or uninfected tumor cells, 7 or 14 days after tumor establishment, no survival increased was observed. In contrast, an increased survival and a sustained immune response was observed when mice were prevaccinated and then subjected to an intracranial rechallenge of the same tumor. In contrast, growth of syngenic, but unrelated, H6 hepatocellular tumor cell line was not affected by vaccination, thus suggesting a specific immunity to Neuro-2a tumors. Indeed, spleen mononuclear cells from vaccinated mice were significantly more cytotoxic to Neuro-2a tumor cells than spleen cells from control mice. Thus, the authors concluded that this vaccine produces a durable and specific immunization in a preclinical model of intracranial tumor [63].

Chakrabarti and coworkers have demonstrated that knock-down of inhibitor of differentiation protein 2 (Id2-kd) in Neuro-2a murine NB cell line rendered these cells immunogenic. Thus, the latter cells (Id2-kd Neuro-2a) grew aggressively in immune-compromised hosts, whereas were rejected by immunocompetent mice, that developed immunity against wild-type Neuro-2a cells. Moreover, they demonstrated that therapeutic vaccination with Id2-kd Neuro-2a cells inhibited tumor growth in established NB tumors, and such antitumor effect was stronger when combined with checkpoint inhibitors. Finally, they have demonstrated, by immune cell depletion, that both CD8^+^ and CD4^+^ T cells are required to establish antitumor immunity and they observed an increased number of IFN-*γ* producing CD8^+^ T cells and infiltration of cytotoxic CD8^+^ T cells within the tumor upon vaccination. These data suggested that downregulation of Id2-kd combined with checkpoint inhibitors may represent a novel therapeutic strategy for NB patients [64].

Another interesting vaccination strategy is based on the use of attenuated Salmonella typhimurium (SL7207) as DNA carrier. It has been demonstrated that oral administration of Salmonella carrying survivin DNA induced a strong cellular anti-NB immune response in a syngeneic mouse model of NB. Furthermore, such antitumor response was greater than that achieved using gene gun application or injection of lentivirally transduced bone marrow-derived dendritic cells (DCs). The limited side effects and the observed antitumor response suggested that this therapeutic strategy may be translated into a clinical application for NB patients [65]. A similar approach has been tested by Fest and coworkers, who have tested a survivin minigene DNA vaccine administered using SL7207 as DNA carrier. They have demonstrated a prophylactic effect, with a reduced tumor growth and metastasis level, and also a therapeutic effect, with eradication of established tumor in more than 50% of treated mice. In both cases, antitumor activity was related to the induction of antitumor CD4^+^ and CD8^+^ mediated immune response, with an increased tumor cell lysis and production of proinflammatory cytokines [66]. Another DNA vaccination strategy was based on the use of plasmids encoding for human tyrosine hydroxylase (hTH), that is expressed at high levels by NB samples, independently of the clinical stage. Three different formulations have been tested for their prophylactic and therapeutic efficacy to suppress tumor growth and metastasis of NXS2 murine NB cells in syngeneic A/J mice: hTHcDNA, hTH minigene, and hTHcDNA in combination with the proinflammatory cytokine IL-12. It has been demonstrated that TH DNA vaccination eradicated established primary tumors and inhibited metastasis formation, and such antitumor effect was not enhanced by IL-12. Depletion of CD8^+^ T cells abrogated anti-NB response, thus suggesting the involvement of tumor-specific CTLs. Finally, surviving mice rechallenged with the same tumor displayed a reduced primary tumor growth, thus demonstrating the induction of a memory immune response. Collectively, these data may open new perspectives for novel and effective vaccination strategies for NB patients [67].

Gil et al. analyzed the therapeutic effect of DCs expressing a CD166 cross-reactive mimotope of the GD2 ganglioside 47-LDA in lymphodepleted NXS2 NB tumor-bearing syngeneic mice. Tumor-specific T cells were adoptively transferred in mice that were subsequently vaccinated with DCs. The 47-LDA mimotope was presented to DCs either as a linear peptide or as a fusion protein with the murine IgG2a Fc fragment (47-LDA-Fcgamma2a). The latter formulation was more effective in the induction of antitumor immune responses [68].

At the present time, 12 clinical trials have been carried out using tumor vaccine for NB patients (11 are competed and one is still recruiting, http://www.clinicaltrials.gov).

#### 2.1.4. Adjuvant Immunotherapy

One of the most interesting approach used to harness the immune system to fight cancer is represented by the use of TLR9 agonists, such as synthetic oligonucleotides containing unmethylated CpG motifs (CpG ODNs). Several years ago, it has been demonstrated that CpG ODNs, through their recognition by TLR9-expressing cells (mainly B lymphocytes, macrophages, and plasmacytoid dendritic cells) trigger the activation of an innate immune response [69]. Moreover, the encapsulation of CpG ODNs in lipidic particles (*i.e.*, liposomes, lipoplexes, *etc.*) results in increased immunostimulatory effects [70].

We demonstrated that the administration of liposomes targeted to NB cells via GD2 and encapsulating c-myb-specific CpG-containing ODNs resulted in inhibition of tumor growth in NB-bearing mice, finally leading to long-term survival. The obtained antitumor activity was the result of both direct inhibition of cell growth (due to decreased c-myb protooncogene expression) and indirect CpG-dependent immune stimulation (due to NK cell-mediated lysis of tumor cells) [71]. CpG-mediated immune stimulation was initiated by macrophages which through the early secretion of IL-12 triggered the activation of NK cells culminating in tumor cells killing.

Subsequently, we have also demonstrated that a therapy that combines the activation of the immune system through the administration of CpG ODN-containing liposomes and antibodies against IL-10R results in improved therapeutic effectiveness in NB-bearing mice. Indeed, IL-10 is an immune-regulatory cytokine known to suppress macrophages and dendritic cell function. In different mouse models of cancer, it has been demonstrated that IL-10 has a pivotal role in dampening antitumor immune response. The combinatorial approach based on the administration of CpG ODN-containing liposomes and antibodies against IL-10R, thanks to the prolongation of immune stimulation induced by CpG ODNs, gave to increased therapeutic results with respect to single therapy [72].

## 3. Malignant Melanoma

Malignant melanoma is one of the most aggressive cancers worldwide, ranking globally as the sixth most frequently diagnosed cancer. It affects both genders, and its incidence is higher in Caucasian with respect to Black people. Further, it is to be underlined that, with respect to other tumors which incidence has been decreased in the last years, the incidence rate for malignant melanoma is continuously increasing rising from about 84,000 cases in 2008 to 100,000 in 2012 in Europe [73]. Melanoma represents only a small proportion of skin cancers; nevertheless, the mortality associated to malignant melanoma is very high, accounting for 80% of skin cancer-related deaths [74].

The efficacy of treatment strategies is strictly related to stage at diagnosis, and although the progress made in the last years with the introduction of immunotherapy and targeted therapies, the prognosis, for stage IV-affected melanoma patients, still remains very poor due to the development of resistance to novel drugs, rendering extremely urgent the search for new effective treatment strategies [75].

### 3.1. Immunotherapy of Melanoma: Preclinical Studies

Several immunotherapeutic approaches have been tested in preclinical models in the last years (Table 2). These include (1) monoclonal antibodies against both immune checkpoints and tumor-associated antigens, (2) tumor vaccines, and (3) cellular therapy. Some of these approaches have been tested in combination with each other, to enhance the immunogenicity of cancer cells and to increase the effects of the immunotherapeutic treatment.

#### 3.1.1. Monoclonal Antibodies against Both Immune Checkpoints and Tumor-Associated Antigens

In the recent years several antibodies targeting immune checkpoints have been approved by the Food and Drug Administration and have been released on the market (*i.e.*, ipilimumab that target CTL-A4; nivolumab and pembrolizumab that target the PD-1 receptor) [76, 77]. The introduction of immune checkpoint inhibitor antibodies in the clinical practice has dramatically improved the outcome of patients affected by advanced melanoma [76, 78]. Nevertheless, research is still searching for new approaches that could be beneficial for treatment of high-stage melanoma patients.

Several studies are aimed to increase the susceptibility of tumor cells to immunotherapy. In this line, it has been demonstrated that topoisomerase I (Top-1) inhibitor irinotecan (MM-398) is able to increase the sensitivity of melanoma cell lines from patients to autologous T cell-mediated cytotoxicity. The authors tested MM-398 in combination with anti-PD-L1 or anti-PD1 mAb *in vivo*, using syngenic mouse models of melanoma. They demonstrated that combined treatment is more effective in the control of tumor growth than MM-398 administered alone. Accordingly, a prolonged survival was observed in mice subjected to combined treatment as compared to mice treated with MM-398 alone [79]. Such effect was mediated by the overexpression of TP53INP1, since knock-down of *TP53INP1* gene completely abrogated T cell-mediated killing of Top1 inhibitor-treated melanoma cells.

Another study combined checkpoint blockade with mAbs targeting tumor antigens, to overcome the escape of tumor cells from the action of immune effector cells. The authors demonstrated that the tumor antigen-targeting mAb TA99, specific for the melanosomal polypeptide gp75 (the most abundant glycoprotein synthesized by pigmented melanocytes and melanomas) [80], increased the fraction of CD4^+^ and CD8^+^ T cells in the tumor microenvironment in B16F10 melanoma mouse model, as compared to mice treated with isotype control. These data suggested that monotherapy with TA99 resulted in a strong immune modulation within the tumor. However, the efficacy of this treatment was limited by the recruitment of Tregs and other immunosuppressive cell subsets that leads to immune escape of tumor cells. They demonstrated that anti-PD-1 treatment of mice, at the time of tumor emergence, restored the effector functions of T cells, NK cells, and *γδ* T cells, leading to the inhibition of tumor progression and extension of survival. These data confirmed that combination of checkpoint blockade and immunotherapy may represent a promising approach for melanoma patients [81].

In another study, the ability of pan-histone deacetylase (HDAC) inhibitor AR42 and sodium valproate to alter the immunogenicity of melanoma cells has been tested *in vitro* and *in vivo*. Melanoma cells treated with HDAC inhibitors downregulated the expression of HDAC proteins, PD-L1, PD-L2, and ornithine decarboxylase and upregulated HLA-A molecules on their surface. Accordingly, AR42 or sodium valproate increased antitumor efficacy of an anti-PD-1 antibody and of an anticytotoxic T lymphocyte antigen 4 (CTLA4) mAb in mice carrying B16F10 melanoma cells. The same results have been obtained with the multikinase inhibitor pazopanib. Animals treated with HDAC inhibitor + anti-PD-1 antibody displayed increased levels of CCL2, CCL5, CXCL9, and CXCL2 in plasma, thus suggesting that the combined treatment increased the infiltration of activated T cells, M1 macrophages, neutrophils, and NK cells. These data support the use of HDAC inhibitors in combination with checkpoint blockade immunotherapy for the treatment of melanoma patients [82].

Since cancer immunotherapy based on immune checkpoint blockade remains ineffective in many patients due to tumoral resistance, Zhu and coworkers used an autochthonous TiRP melanoma model, which recapitulates the tumoral resistance signature observed in human melanomas and is resistant to immunotherapeutic strategies based on checkpoint blockade, cancer vaccines, or adoptive T cell therapy. In this model, CD8^+^ T cells are recruited within the tumor, but these cells undergo apoptosis. This occurs through the action of myeloid derived suppressor cells (MDSC), that are increased in TiRP tumors, and expressed high levels of Fas-ligand, thus inducing apoptosis of T cells through the interaction with Fas in T cells. Indeed, T cells are rescued from apoptosis by interrupting Fas/Fas-ligand axis using mAbs specific for these molecules. Thus, this interesting study demonstrated that immunotherapy resistance in melanoma can be overcome by a combined treatment with Fas/Fas-ligand blocking antibodies [83].

In a recent study, Rakhmilevich and colleagues assayed the efficacy of combining innate and adaptive immunotherapeutic approaches in mouse models of melanoma (*i.e.*, B16-GD2 which was transfected to express GD2 and B78, a slow-growing B16 derivative which expresses GD2) [84]. The authors demonstrated that the administration of anti-CD40 mAb (costimulatory receptor belonging to the superfamily of tumor necrosis factor receptor, predominantly expressed by antigen-presenting cells) with CpG ODNs was able to suppress tumor growth in the B78 melanoma model; these results were due to tumor cell killing via activation of macrophages. Moreover, the combination of anti-CD40 and CpG ODNs was also able to activate T cell, due to augmentation of Ag presentation. In the same melanoma models, authors demonstrated that combining immune checkpoint blockade using anti-CTL-A4 and an immunocytokine (IC) consisting of the anti-GD2 Ab 14.18 and IL-2 (14.18-IL-2 IC) induced tumor regression in the B78 melanoma model, resulting in a survival rate of about 40% of tumor-bearing mice. Authors determined that the observed antitumor effects were T cell mediated, because they were not observed in nude mice. Finally, the multicombinatorial approach consisting in the administration of anti-CD40/CpG ODNs and anti-CTL-A4/14.18-IL-2 IC was also assayed. This treatment led to increased antitumor effects with respect to single therapy and can be justified by the cooperation of innate and adaptive immunity [84].

As expected, due to the high incidence of melanoma worldwide, several clinical trials have been performed in the field of immunotherapy. If we focus on studies carried out in stage IV melanoma patients, we can find 106 clinical trials testing the use of monoclonal antibodies targeting both immune checkpoints and tumor-associated antigens, some of them have been concluded, while others are active and/or recruiting patients. Moreover, many of the aforementioned studies are focused on combinatorial approaches (http://www.clinicaltrials.gov).

#### 3.1.2. Tumor Vaccines

Another strategy to improve antitumor immune response is to target immunosuppressive cell subsets in the tumor microenvironment. In this respect, Namdar and colleagues tested the efficacy of prophylactic Foxp3 DNA/recombinant protein vaccine, that has been previously demonstrated to promote immunity against Tregs in tumor-free conditions, in a B16F10 melanoma model. They have demonstrated that this vaccine downregulated Tregs in spleen and tumor microenvironment, and such effect was associated to a reduction of MDSC in both compartments. Moreover, Foxp3 vaccine significantly reduced arginase-1- (Arg-1-) induced nitric oxide synthase (iNOS) and production of reactive oxygen species (ROS) and suppressed MDSC activity. The concomitant reduction of Tregs and MDSC reverted the immunosuppression within the tumor and increased the local production of IFN-*γ* and CTL-mediated antitumor immune response. Vaccinated mice showed a reduced tumor growth and a prolonged survival as compared to control mice, thus suggesting that blocking of immunosuppressive cell subsets may represent a promising therapeutic tool for melanoma patients [85].

Sorensen and colleagues demonstrated that in a B16F10 melanoma model, the therapeutic vaccination using an adenoviral vector expressing a tumor-associated antigen (GP33-41) statistically delayed the tumor growth of B16F10 melanoma which expresses the same antigen, although not leading to a complete regression. In this model, the authors further demonstrated that, in vaccinated mice, the combination of anti-CD40 mAb and immune check point blockade using the anti-CTL-A4 mAb was able to delay tumor growth, resulting in complete regression and long-term survival of tumor-bearing mice. The antitumor effect observed was higher with respect to either treatment individually applied and was due to increased CD8^+^ T cell response against the abovementioned tumor-associated antigen [86].

It is to be underlined that a huge amount of clinical trials have been carried out using tumor vaccine in melanoma; a lot of them are completed and some are still active and/or recruiting patients, highlighting the great expectation that clinicians place into them (http://www.clinicaltrials.gov). Moreover, dendritic cell vaccine is currently used as melanoma therapy [87–89].

#### 3.1.3. Cellular Therapy

One of the major limitation that cellular therapy can face to is represented by tumor-induced immune suppression that include among others also immune cells such as Tregs and MDSC [90–92]. In this respect, Kodumudi and colleagues demonstrated that in a murine model of melanoma, the total body irradiation-induced lymphopenia followed by docetaxel treatment improved the efficacy of adoptive T cell transfer and dendritic cell immunotherapy in melanoma-bearing mice, inducing a significant reduction in tumor growth finally resulting in enhanced survival. Tumor regression correlated with increased CTL activity and persistence of adoptively transferred T cells [93].

A novel approach based on cellular therapy was assayed in preclinical setting by Hu and colleagues [94]. In this study, authors developed a humanized mouse model that permitted the production of human T cells engineered to express the melanoma antigen MART-1-specific TCR. The adoptive transfer of MART-1 TCR^+^ CD8^+^ T cells in tumor-bearing mice enabled the efficient killing of melanoma cells in an antigen-specific manner, finally resulting in protection of metastatic melanoma and prolonged survival rate. Moreover, the coadministration of MART-1 TCR^+^ CD8^+^ T cells and IL-15 led to improved antitumor effects.

As reported above for other immunotherapeutic approaches, also for cellular therapy, several clinical trials have been carried out (http://www.clinicaltrials.gov).

## 4. Conclusions

We have here summarized recent data regarding novel immunotherapeutic approaches that have been tested in preclinical models of human NB and MM. These studies have demonstrated that these novel strategies based on the use of monoclonal antibodies and cytotoxic cells specific for tumor-associated antigens may represent promising therapeutic tools. Moreover, the combination of these approaches with adjuvant therapies or checkpoint inhibitors increases antitumor efficacy. Some of these strategies have been already included in clinical treatment settings. The translation of studies that are still at the preclinical phase to the clinic will be pivotal to evaluate the real effectiveness of these therapeutic approaches on cancer patients.

## Figures and Tables

**Table 1:** Summary of novel immunotherapeutic strategies for neuroblastoma patients.

Target	Therapeutic strategy	Reference
GD2	CAR T cells	[24]
GD2	CAR T cells + bevacizumab	[25]
GD2	ScFv CAR T cells	[26]
NY-ESO-1	T cells with high-affinity transgenic T cell receptor specific for NY-ESO-1	[30]
GD2	Anti-GD2 mAb + TCR*γδ* T cells + zoledronic acid	[36]
Phosphoantigens	V*γ*9V*δ*2 lymphocytes + zoledronic acid	[35]
PRAME	PRAME-specific HLA-A2^+^ CTLs + NK cells	[40]
ALK	ALK-specific HLA-A2^+^ CTLs	[43]
Ligands of NCR	Polyclonal NK cells	[47]
GD2	Anti-GD2 mAb + anti-PD-L1 mAb	[51]
GD2	Anti-GD2 mAb + TGF*β*R1 inhibitor	[52]
PD-1/PD-L1	Anti-PD-1 mAb + anti-PD-L1 mAb + anti-CD4 mAb	[57]
CD171	CAR T cells	[61]
GPC2	Anti-GPC2 mAb conjugated with drugs	[62]
Unknown	Vaccination with tumor cells infected with IL-12-expressing HSV-1 virus	[63]
Unknown	Vaccination with tumor cells with a knock-down of *Id2* gene	[64]
Survivin	Vaccination with Salmonella typhimurium carrying survivin DNA	[65, 66]
Tyrosine hydroxylase	Vaccination with plasmids encoding for human tyrosine hydroxylase	[67]
GD2	Vaccination with DC expressing a CD166 cross-reactive mimotope of GD2	[68]
c-myb	GD2-targeted liposomes encapsulating c-myb-specific CpG-containing ODNs	[71]
c-myb	GD2-targeted liposomes encapsulating c-myb-specific CpG-containing ODNs + anti-IL10R mAb	[72]

**Table 2:** Summary of novel immunotherapeutic strategies for melanoma patients.

Target	Therapeutic strategy	Reference
Topoisomerase I	Topoisomerase I inhibitor + anti-PD-L1 or anti-PD1 mAbs	[79]
Polypeptide gp75	Anti gp75 mAb + anti-PD1 mAb	[81]
Histone deacetylase (HDAC)	Pan-HDAC inhibitors + anti-PD1 mAb	[82]
Fas	Fas/Fas-ligand blocking mAbs	[83]
CD40	Anti-CD40 mAb + CpG ODNs	[84]
CTLA-4 and GD2	Anti-CTL-A4 mAb + anti-GD2 mAb conjugated with IL-2	[84]
Regulatory cells	Foxp3 DNA/recombinant protein vaccine	[85]
GP33-41 antigen	Adenoviral vector expressing GP33-41 tumor-associated antigen	[86]
Unknown	Docetaxel + adoptive T cell transfer + DC immunotherapy	[93]
MART-1	MART-1-specific CD8^+^ T cells	[94]
